# Variability in the management of healthy short youth following GH stimulation testing

**DOI:** 10.3390/endocrines7030037

**Published:** 2026-07-07

**Authors:** Adda Grimberg, Victoria A. Miller, Morgan P. Snyder, Elizabeth A. Friedrich

**Affiliations:** 1 Dept of Pediatrics, Perelman School of Medicine, University of Pennsylvania, Philadelphia, PA, USA; 2 Division of Endocrinology and Diabetes, Children’s Hospital of Philadelphia, Philadelphia, PA, USA; 3 Leonard Davis Institute of Health Economics, Perelman School of Medicine, University of Pennsylvania, Philadelphia, PA, USA; 4 Craig Dalsimer Division of Adolescent Medicine, Children’s Hospital of Philadelphia, Philadelphia, PA, USA

**Keywords:** short stature, growth hormone, pediatric, stimulation tests, treatment, clinical decision making

## Abstract

**Background/Objectives:**

Recent Delphi survey of endocrinologists revealed low consensus regarding the diagnosis of pediatric growth hormone deficiency (GHD). Thus, we sought to describe the various trajectories undertaken by healthy 8-14 year old youth in the 2 years following testing for GHD at a single major pediatric academic institution.

**Methods:**

Electronic health records were reviewed for the current analysis from healthy 8-14 year old participants enrolled in a prospective longitudinal observational study of parent and youth characteristics associated with youth quality of life and self-esteem over a two-year period following growth hormone (GH) stimulation testing. Participants were grouped according to their peak GH concentration on testing (<7, 7-10, and ≥10 ng/ml), and outcomes included treatment (or not) with GH or other growth-altering hormonal treatments.

**Results:**

Of the 115 participants, 27 (23%) had peak GH <7 ng/ml, 27 (23%) 7-10 ng/ml, and 61 (53%) peaked ≥10 ng/ml. Across the 3 groups, some patients were not offered GH treatment, some were offered yet did not pursue treatment, and some were offered and treated – with further variance provided by GH treatment interruptions, early cessation *vs* continued GH treatment, delayed GH treatment start, and treatment with other agents (testosterone, gonadotropin releasing hormone agonist, or aromatase inhibitor) either in lieu of or in addition to GH.

**Conclusions:**

Even within the network of a single academic institution, variability is evident in the management of healthy 8-14 year old short youth following GH stimulation testing.

## 1. Introduction

As we approach the 70^th^ anniversary of the first human treatment with human growth hormone (GH) [1], diagnostic and treatment dilemmas persist [2]. This was laid bare by the Growth Hormone Research Society’s recent Delphi survey of 43 endocrinologists (18 who worked with children and transition patients, and 25 who worked with transition and adult patients) from 14 countries and an average clinical experience of 26 years [3]. After 2 rounds of rating their degree of agreement with 61 statements regarding the diagnosis of GH deficiency (GHD), consensus (predefined as ≥ 80% of panelists uni-directionally rating their agreement with each statement as ≥ 5 or ≤ 3 on the 7-point Likert-type scale) was reached on 17 of 29 (59%) pediatric statements and 28 of 32 (88%) adult statements. The statements addressed 4 categories: whom to test for GHD; when to test for GHD; how to test for GHD; and how to interpret test results [3].

Generally, pediatric GHD is considered in youth who present with worrisome anthropometrics. Comprehensive history and physical examinations are performed, bone age radiographs are used to assess the extent of skeletal maturation, and laboratory tests are obtained to screen for systemic causes of growth failure that call for non-GH treatments, such as celiac disease or hypothyroidism. After abnormally low circulating concentration of insulin-like growth factor (IGF)-1 and/or IGF binding protein (IGFBP)-3 raises the possibility of GHD, the diagnosis is confirmed (or not) by GH stimulation tests. Upon diagnosis of GHD, magnetic resonance imaging evaluates the pituitary structurally. Because current GH assays are more sensitive than the historical ones used during GH stimulation test development, lowering the diagnostic threshold from a peak GH level of 10 ng/ml to 7 ng/ml has been proposed as a way of reducing GH over-treatment of youth with “false failing” results on the stimulation tests [4]. Hence, GH test results can be categorized as GHD (<7 ng/ml), normal/GH replete (≥10 ng/ml) and in-between/gray area (7-10 ng/ml).

We recently completed a longitudinal study with about 27 months of observation after GH stimulation testing of healthy 8-14 year old youth, whose clinical care was determined by their endocrinologists independent of study participation. In light of the aforementioned controversies in the diagnosis of GHD, we therefore sought to describe the various trajectories undertaken by patients undergoing evaluation for GHD at a single major pediatric academic institution with over 50 endocrine practitioners (attending and fellow physicians, as well as clinical nurse practitioners) who see patients in a network of clinics in urban and non-urban settings in Pennsylvania and New Jersey.

## 2. Materials and Methods

The goal of the overall longitudinal study was to identify parent and youth characteristics associated with youth quality of life (QoL) and self-esteem over a two-year period following GH stimulation testing of healthy short youth. From May 2019 to May 2023, weekly reports of the Children’s Hospital of Philadelphia (CHOP) Day Medicine Unit schedule were previewed to identify 8-14 year old youth referred by their treating endocrinologists, according to their clinical judgment, for GH stimulation testing. Exclusion criteria were: developmental delay or cognitive impairment that would impede completion of the study questionnaires and interviews, a physical disability or life-threatening medical condition that required accommodations for activities of daily living or daily treatment for at least 6 months of the preceding year, or a past psychiatric hospitalization. One primary caregiver per family, hereafter referred to as parent, was eligible to participate, and all participants spoke English.

Recruitment letters for a prospective, observational, longitudinal study (6 visits over approximately 27 months) were sent to 421 parent-youth dyads identified from the GH testing schedule; the research team was able to contact 319 by phone. Of these, 202 (63%) parents expressed interest in participating. Upon callback for inclusion/exclusion criteria screening and enrollment, 133 (66%) were reached and 120 (59%) dyads enrolled in the study (9 deemed ineligible, 4 youth declined). Consent and verbal permission/assent occurred during the screening and enrollment call, per IRB-approved protocol.

The final sample for the current analysis consisted of 115 dyads, as 3 of the 120 enrolled were later deemed ineligible and 2 withdrew from the study before Visit 2. 112 parents and 112 youth (112 dyads) completed Visit 2; 104 parents and 102 youth (100 dyads) completed Visit 3; 107 parents and 101 youth (99 dyads) completed Visit 4; 104 parents and 102 youth (99 dyads) completed Visit 5; and 100 parents and 102 youth (96 dyads) completed Visit 6.

For Visits 1 and 2, parents and youth independently completed questionnaires with trained research staff over the phone or prior to March 2020 (before COVID-19 precautions), in person during the GH stimulation testing. Questionnaires at all subsequent visits were completed by youth over the phone and by parents electronically. Visit 1 questionnaires were required to be completed before the GH stimulation tests concluded, but no more than one month before testing. Visit 2 occurred within 3 months of the GH testing date, with visits 3-6 approximately 6, 12, 18, and 24 months after Visit 2. Participants were compensated for each visit with gift cards.

The questionnaires assessed demographics, parent characteristics (perceived threat to child in current and future environments; mastery-oriented goals for the child; autonomy support), youth characteristics (coping skills; perceived social support), QoL and self-esteem, as previously reported [5]. Pertinent to the current study, electronic health records were reviewed for clinical data: sex-adjusted mid-parental height z-score, height (both absolute measurement and age- and sex-specific z-score; at every visit), height deficit from mid-parental height z-score, weight and BMI z-score (every visit), Tanner stage (every visit), bone age z-score, peak GH concentration on stimulation testing, and GH (or other) treatment status (every visit). Interpretation of the GH test results and all clinical care were determined by the youths’ endocrinologists, unaffected by participation in the study.

Data preparation and all statistical analyses were performed using SAS^®^ 9.4 (SAS, Cary, NC) and Microsoft Excel (Microsoft Corp., Redmond, WA). Participants were grouped based on their peak GH concentration on testing (<7, 7-10, or ≥10 ng/ml). Demographics and clinical variables were summarized using standard descriptive statistics including frequencies and percentages for categorical variables and mean, standard deviation (SD) and range for continuous variables.

This study was approved by the CHOP Institutional Review Board (protocol no. 18-015328).

## 3. Results

### 3.1. Participants

Characteristics of the 117 eligible youth who completed Visit 1 are summarized in Table 1. Youth were 68% male (n= 80) with a mean age of 11.49 years (SD= 1.98), mean height z-score of -2.30 (SD= 0.66), and mean bone age of 10.30 (SD= 2.48). Forty-six youth (42%) were in early puberty while the rest (n=64; 58%) were still prepubertal. Their participating parents were primarily female (n= 104; 89%) and White (n= 103; 88%) and had achieved a high education level (n= 62; 53% with more than a 4-year college degree).

The 115 youth included in the present analysis saw 34 unique pediatric endocrinology providers around the time of Visit 1. Each of these providers saw a range of 1-25 youth participants (mean= 3.38; median= 2). Twenty-four of the youth saw an additional 1-2 CHOP providers over the course of the study.

### 3.2. Peak GH < 7 ng/ml

**Figure 1.**
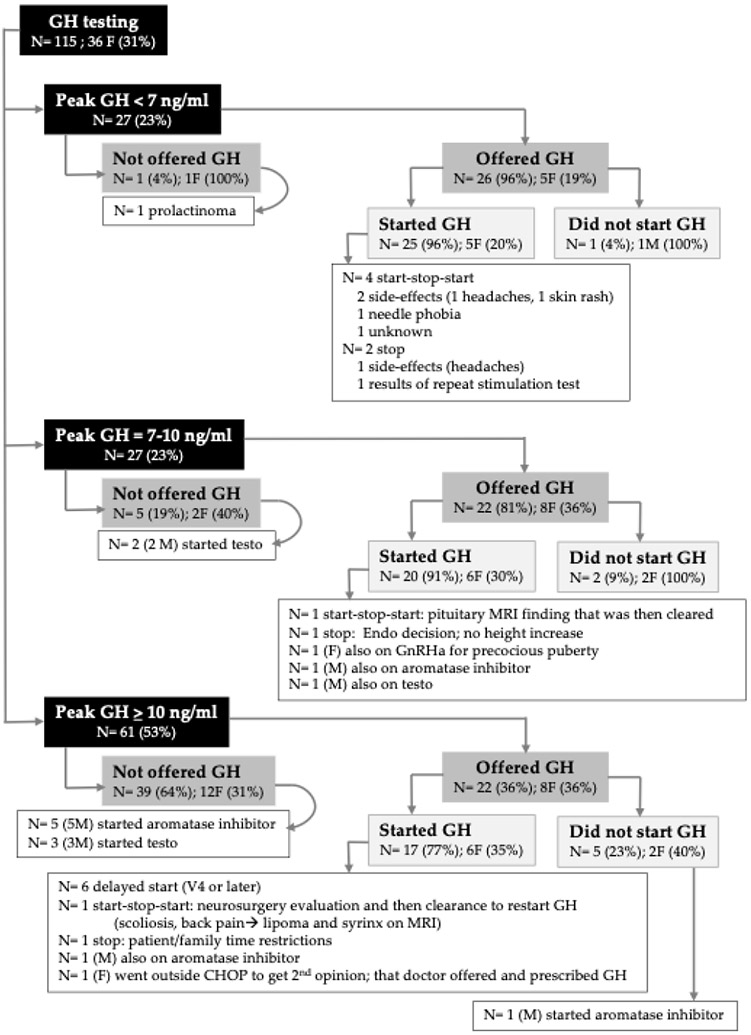
Flow diagram of the various trajectories undertaken by patients based on their peak GH concentration on stimulation testing. testo = testosterone, GnRHa = gonadotropin releasing hormone agonist

Of the 115 patients, 27 (23%) had a peak GH of < 7 ng/ml (see Figure 1 and Table 2). Of these, 1 (4%) was not offered GH treatment due to prolactinoma, and 26 (96%) were offered GH. Of those who were offered GH treatment, 1 (4%) did not start GH, and 25 (96%) started GH. Of those who started, 4 stopped and then restarted GH (2 due to side effects, 1 due to needle phobia, and 1 for unknown reason). Additionally, of those who started, 2 completely stopped GH prior to the end of the 2-year observation window (1 due to side effects; 1 due to results of a repeat GH stimulation test). The remainder were still receiving GH by the last study visit.

### 3.3. Peak GH = 7-10 ng/ml

Of the 115 patients, 27 (23%) had a peak GH of 7-10 ng/ml (see Figure 1 and Table 2). Of these, 5 (19%) were not offered GH treatment; 2 of these boys started testosterone treatment. Of the 27, 22 (81%) were offered GH. Of those who were offered GH treatment, 2 (9%) did not start GH, and 20 (91%) started GH. Of those who started, 1 stopped and then restarted GH (due to concerns about the pituitary on MRI that were subsequently cleared), and 1 completely stopped GH prior to the end of the 2-year observation window (endocrinologist’s recommendation due to lack of height increase in response to treatment). Of those who started GH, 1 girl was also treated with gonadotropin releasing hormone agonist (GnRHa) for precocious puberty, 1 boy was also treated with aromatase inhibitor, and another boy also received testosterone treatment. Nineteen patients were still receiving GH by the last study visit.

### 3.4. Peak GH ≥ 10 ng/ml

Of the 115 patients, 61 (53%) had a peak GH of ≥ 10 ng/ml (see Figure 1 and Table 2). Of these, 39 (64%) were not offered GH; 3 of these boys started testosterone treatment and 5 boys started treatment with an aromatase inhibitor. Of the 61, 22 (36%) were offered GH treatment. Of those who were offered GH, 5 (23%) did not start GH; 1 of these boys started an aromatase inhibitor instead. Of those who were offered GH, 17 (77%) started GH treatment. Of those who started, 6 were considered delayed starts (starting GH treatment at study Visit 4 or later, i.e. over a year after GH stimulation testing), 1 stopped and then restarted GH (due to back-related concerns and need for a neurosurgery evaluation of the spine), 1 completely stopped GH prior to the end of the 2-year observation window (due to patient/family time restrictions), and 1 sought a second opinion from a provider outside of CHOP and was prescribed GH by that provider. Of those who started GH, 1 boy was also treated with aromatase inhibitor. Sixteen patients were still receiving GH by the last study visit.

## 4. Discussion

In summary, even within the network of a single academic institution, variability is evident in the management of healthy 8-14 year old short youth following GH stimulation testing. Across all 3 categories of peak GH results (< 7, 7-10, and ≥ 10 ng/ml), some patients were not offered GH treatment, some were offered yet did not pursue treatment, and some were offered and treated – with further variance provided by GH treatment interruptions, early cessation *vs* continued GH treatment, delayed GH treatment start, and treatment with other agents (notably, testosterone, GnRHa, or aromatase inhibitors) either in lieu of or in addition to GH.

Why should GHD diagnosis and GH treatment be so highly variable, both in the hypothetical (as seen with the low consensus rates on the Growth Hormone Research Society Delphi survey [3]) and in a real-world setting (as seen here)? There are 5 major contributors:

Foremost, we are trying to capture a moving target. GH is secreted primarily in pulses during deep sleep, whose light entrainment develops gradually over the first few months of life [6]. The advent of frequent serial blood sampling techniques enabled measurement of endogenous secretory patterns and circadian rhythms [7], and was itself used for a period of time as a diagnostic test of GHD. However, the pattern of pulses, their amplitude and the mean integrated GH concentration over 24 hours was found to be highly variable even when the same individual was tested on different days/nights during a week’s admission to a research unit wherein diet and activity were kept constant [8]. With such intraindividual variability, let alone interindividual variability, how does one define “normal” and distinguish it from “abnormal”?

Thus, GH stimulation tests were developed as a means of creating a GH pulse during testing and thereby bypassing the complications of circadian rhythmicity. Unfortunately, the multiple GH stimulation tests tried throughout the decades are themselves highly problematical, including poor sensitivity and specificity [9], providing the second factor underlying the variability in GHD diagnosis and GH treatment. When 472 normally growing children were tested by 10 different GH stimulation tests, a high percentage had peak GH levels below the arbitrarily designated diagnostic cut-off, so much so that the authors concluded, “The finding of such low GH responses in normal subjects makes these tests unsuitable for discriminating between normal and GH deficient subjects [10].” Despite this, GH stimulation testing became entrenched as standard of care for want of better alternatives.

A third major contributor to the variability in GHD diagnosis and GH treatment is the lack of standardization, in both assays and testing protocols [11]. While this factor would not directly impact the variability in our single-center study, it does increase variance across the GH literature which in turn contributes to the incomplete clinical confidence in the diagnostic acumen of GH stimulation test results. Normative data for GH stimulation tests were established using polyclonal radioimmunoassays and purified pituitary standards to measure GH, while current GH tests use immunometric assays with monoclonal antibodies and recombinant standards. Methodological differences among assays can involve competitive *vs* sandwich-type designs, the specific antibodies used (monoclonal *vs* polyclonal; different epitope specificity or reactivity), antibody-antigen reaction conditions (equilibrium *vs* nonequilibrium state), effects of the specific matrix used, and differences in the reference standards [12]. Calls for the use of harmonized GH assays with the somatropin standard, IRP IS 98/574, 22k rhGH isoform, which is the current state-of-the-art [13], did not occur until 2011 [14]. While such technical differences might seem picky, they lead to clinically significant issues. For example, comparing 3 GH assays (2 using the same standard, 88/624), repetitive measurement of 699 peak samples from GH stimulation tests yielded a mean difference of 2.7 to 5.1 ng/ml among assays, and misclassification as GHD/GH-sufficient in up to 29% of 132 patients undergoing standardized insulin and arginine testing [15]. Similarly, when samples from 47 stimulation tests were remeasured using 4 different assays, GHD would have been diagnosed in 36% *vs* 15% of the tests depending on the assay used [16]. Compounding the lack of standardized GH assays is the absence of standardized GH stimulation test protocols. While convention has adopted the requirement to fail two out of two stimulation tests to be diagnosed with GHD, as a means of mitigating the high false failure rate [4,13], how the two tests are combined in practice is not evidence-based. Differences in sampling approach can alter results. For example, close sampling during insulin stimulation testing of 76 patients would have “passed” (i.e. peak GH ≥ 7 ng/ml) an additional 11 (14%) patients if sampling began with intravenous catheter placement and not 30 minutes later with administration of insulin [17], as is commonly performed.

The fourth major contributor to variability in GHD diagnosis and GH treatment is perhaps most readily evident from this study’s results. Due to the problems with GH stimulation testing, the GH Guidelines of the Pediatric Endocrine Society strongly recommended “against reliance on GH provocative test results as the sole diagnostic criterion of GHD [12].” GHD is more of a gestalt diagnosis, and test results can be improved by careful patient selection and mitigation of potential confounding factors [4,13,18-20]. Some methods proposed include sex hormone priming prior to GH testing to reduce misdiagnosis of GHD in adolescents with constitutional delay of growth and puberty, considering the blunting effect of obesity on GH secretion [21], observing patients with equivocal test results for additional data (growth, weight/BMI and IGF-I/IGFBP-3 trends) prior to starting GH treatment, trying alternative treatments (like testosterone), and pausing GH treatment for reassessment/retesting. Such nuanced approaches are not only discussed by international experts in the literature [4,13,18-20], but play out in real-world clinical practice as seen in Figure 1.

Finally, the fifth major contributor to the variability in clinical practice is the underlying social aspects: the shared decision-making process involving both the patient-family and clinician, the societal preferences for height and how much society (and/or the family) is willing to pay for pharmacological intervention. While we like to think of medical tests as scientifically sound and providing “the answer” that logically and directly informs next steps in clinical management, the real-world process is more complicated. Evidence shows that the degree of parental concern influences rates of both referral by primary care clinicians and prescription of GH by endocrinologists [22-24], independent of objective measures of the youth’s growth. Parents consider multiple factors in their height-related medical decision-making, that span treatment characteristics (proven efficacy and safety), health concerns and social functioning (both in childhood and projected into adulthood) [25,26]. Heightism refers to the pervasive societal prejudice against short stature and preference for tall stature as an association of markers of social success [27]. Heightism not only influences the growth-related shared decision-making process about a given patient, but growth-related clinical care at large. Desire for pharmacologic height enhancement as a means of improving QoL by mitigating heightism-related distress [27] has helped fuel the expansion of pediatric GH treatment in the USA by nearly three-fold from 2001 to 2016 [28]. However, insurance providers have increasingly stopped covering GH treatment for idiopathic short stature [29], likening it to medical cosmetology. For many youth, the only distinction between being diagnosed with GHD *vs* idiopathic short stature is their peak GH concentration on stimulation testing. Thus, what happens in the idiopathic short stature field colors, consciously or subconsciously, how stringently people want to define GHD. Heightism and financial factors also drive health care consumerism, such as “doctor shopping” (families seeking opinions from different endocrinologists until finding one amenable to prescribing GH, like a patient in our sample), and the willingness to turn to alternative treatments even if off-label, such as aromatase inhibitors [30] (also seen in our sample). Unfortunately, increased subjectivity of test interpretation and medical decision-making increases vulnerability to disparities in care. Gender, racial, ethnic and socioeconomic disparities are evident in all steps of the medical management of short stature/growth failure in youth [31], including that of the GH stimulation test specifically [32, 33].

Generalizability of the findings of this study is limited by its sampling of patients from a single major academic center and its focus on healthy short youth age 8-14 years for purposes of the primary QoL study from which the data were culled.

## 5. Conclusions

In conclusion, variability is evident in the medical management of healthy short youth following GH stimulation testing, likely driven by multiple factors including the challenges of capturing GH secretory patterns, the limitations of our current tests, and underlying social and financial influences. Until additional research develops better diagnostic tests for GHD, real-world clinical practice mirrors published expert advice in attempting to improve testing precision and subsequent decision-making by allowing for nuanced considerations of the presentation and course of the individual patient.

## Figures and Tables

**Table 1.** Characteristics of the eligible participants who completed Visit 1^1^ (N= 117).

Youth Characteristics	Mean (SD), range or n (%)
Sex (male)	80 (68.38)
Age (yrs)	11.49 (1.98), 8-14
Race	
White	93 (79.49)
Black/African American	8 (6.84)
Other/More than one race	9 (7.69)
Asian	7 (5.98)
Ethnicity (Hispanic)	10 (8.62)
Height (cm)	134.28 (12.10), 109.90-156.60
Height z-score	−2.30 (0.66), −4.20 to −0.50
Weight z-score	−1.63 (1.12), −4.88 to 1.54
BMI z-score	−0.35 (1.07), −4.80 to 2.13
Tanner stage	
1	64 (58.18)
2 or 3	46 (41.81)
4 or 5	0 (0)
Bone age (y)	10.30 (2.48), 5.00-16.00
Height deficit from mid-parental height z-score	−2.17 (1.11), −5.31-0.40

1 Data were missing/refused for: youth ethnicity (n= 1), Tanner stage (n= 7), and bone age (n= 14).

**Table 2.** Youth age, sex, and height z-score by GH stimulation test result and whether patient was offered GH (N= 115).

	Mean (SD) or n (%)					
	< 7 ng/ml (n= 27)		7-10 ng/ml (n= 27)		≥ 10 ng/ml (n= 61)	
Youth Characteristics	Not offered GH (n= 1)	Offered GH (n= 26)	Not offered GH (n= 5)	Offered GH (n= 22)	Not offered GH (n= 39)	Offered GH (n= 22)
Age (years)	14.34 (n/a)	12.26 (1.90)	13.04 (2.16)	12.29 (1.78)	11.82 (2.20)	11.51 (1.80)
Sex						
Female	1 (4)	5 (19)	2 (40)	8 (36)	12 (31)	8 (36)
Male	0	21 (81)	3 (60)	14 (64)	27 (69)	14 (64)
Height z-score	−2.28 (n/a)	−1.95 (0.51)	−1.69 (0.60)	−1.89 (1.25)	−2.19 (0.60)	−2.27 (0.69)

## Data Availability

The raw data supporting the conclusions of this article will be made available upon reasonable request.

## Abbreviations

The following abbreviations are used in this manuscript:

CHOP: Children’s Hospital of Philadelphia
GH: growth hormone
GHD: growth hormone deficiency
GnRHa: gonadotropin releasing hormone agonist
IGF: insulin-like growth factor
IGFBP: IGF binding protein
QoL: quality of life
Testo: testosterone
